# Arginine and Citrulline and the Immune Response in Sepsis

**DOI:** 10.3390/nu7031426

**Published:** 2015-02-18

**Authors:** Karolina A.P. Wijnands, Tessy M.R. Castermans, Merel P.J. Hommen, Dennis M. Meesters, Martijn Poeze

**Affiliations:** 1 Department of Surgery, NUTRIM School for Nutrition, Toxicology and Metabolism, Maastricht University Medical Center, Maastricht 6200 MD, The Netherlands; E-Mails: d.meesters@maastrichtuniversity.nl (D.M.M.); m.poeze@maastrichtuniversity.nl (M.P.); 2 Department of Surgery, Maastricht University Medical Center, Maastricht 6200MD, The Netherlands; E-Mails: TMR.castermans@maastrichtuniversity.nl (T.M.R.C.); m.hommen@student.maastrichtuniversity.nl (M.P.J.H.)

**Keywords:** arginine, citrulline, nitric oxide, sepsis, immunity

## Abstract

Arginine, a semi-essential amino acid is an important initiator of the immune response. Arginine serves as a precursor in several metabolic pathways in different organs. In the immune response, arginine metabolism and availability is determined by the nitric oxide synthases and the arginase enzymes, which convert arginine into nitric oxide (NO) and ornithine, respectively. Limitations in arginine availability during inflammatory conditions regulate macrophages and T-lymfocyte activation. Furthermore, over the past years more evidence has been gathered which showed that arginine and citrulline deficiencies may underlie the detrimental outcome of inflammatory conditions, such as sepsis and endotoxemia. Not only does the immune response contribute to the arginine deficiency, also the impaired arginine *de novo* synthesis in the kidney has a key role in the eventual observed arginine deficiency. The complex interplay between the immune response and the arginine-NO metabolism is further underscored by recent data of our group. In this review we give an overview of physiological arginine and citrulline metabolism and we address the experimental and clinical studies in which the arginine-citrulline NO pathway plays an essential role in the immune response, as initiator and therapeutic target.

## 1. Introduction

Sepsis and associated inflammatory conditions, such as bacteremia and endotoxemia; a condition with increased presence of lipopolysaccharide (LPS), the essential component of the outer membrane of Gram-negative bacteria, are associated with arginine deficiency [1,2,3,4]. This arginine deficiency is suggested to be the result of a decreased arginine uptake and an impaired arginine *de novo* synthesis from citrulline, in combination with an enhanced arginine catabolism by the upregulation of arginase and the inflammatory nitric oxide synthase (iNOS; NOS2) in the immune response. As a result, strategies to improve the arginine availability during inflammatory conditions have gathered widespread attention over the past decades. However, this does not necessary mean that arginine deficiency occurs universally in all inflammation or sepsis related illnesses. It is beyond the purpose of this review to determine whether arginine deficiency is present in all these conditions. The purpose of this review is to provide an overview of the recent literature that discusses the presence of arginine deficiency during sepsis and endotoxemia, and to discuss the recent studies on improving the arginine and citrulline availability in relation to the immune response. The focus of this review is on the role of the arginine-citrulline nitric oxide (NO) metabolism during these inflammatory conditions, such as endotoxemia and sepsis. In addition, strategies to influence the arginine and citrulline availability in endotoxemia will be discussed. Furthermore, to understand the role of arginine and citrulline and the immune response during inflammatory conditions such as sepsis, insight in the normal physiological processes of arginine and citrulline and the relevant enzymes that maintain this metabolism is essential. Therefore, the important relevant metabolic pathways of arginine in health will be highlighted.

## 2. Arginine during Physiological Conditions

Arginine is an amino acid that can be derived from dietary intake (approximately 4–6 g of arginine per day) [5], from *de novo* synthesis from citrulline (10%–15% of the total arginine production) [6,7,8,9,10,11] and through protein breakdown (approximately 80%) [7,10]. Arginine is poorly absorbed in the intestine, with the jejunum as major absorption site, and exhibits a significant liver uptake [6,12]. The synthesized arginine from citrulline accounts for 60% of the *de novo* whole-body arginine synthesis, however this only represents 5%–15% of the total circulating arginine. This indicates that most of the plasma arginine is derived from proteolysis and food intake [7,8,9,13,14,15].

**Figure 1:**
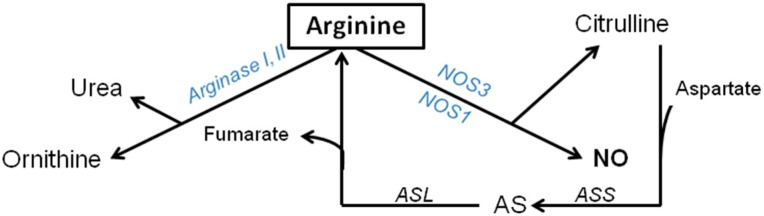
Arginine-citrulline-NO metabolism during physiological conditions. Abbreviations: NO, nitric oxide; NOS3, endothelial nitric oxide synthase; NOS2, inducible nitric oxide synthase; ASS, argininosuccinate synthetase; ASL, argininosuccinate lyase; AS, argininosuccinate.

Arginine plays a key role in several metabolic pathways; however it is beyond the purpose of this review to discuss all the pathways of arginine during physiological conditions (see references [14,16,17,18,19] for reviews). In this review only the arginine *de novo* synthesis-regulated anabolic and the arginase and the NOS enzymes-regulated catabolic pathways of arginine will be discussed (Figure 1), since these pathways play an essential role during inflammatory conditions.

### 2.1. Arginine de novo Synthesis; Conversion of Citrulline into Arginine by Argininosuccinate Synthetase and Argininosuccinate Lyase

Arginine can be produced from the conversion of citrulline by the combined action of the cytosolic enzymes argininosuccinate synthetase (ASS) and argininosuccinate lyase (ASL) [9,10,20,21,22,23,24]. Citrulline, the limiting factor in the arginine *de novo* synthesis [9], exhibits a low dietary intake (approximately 13% of total arginine) [25]. However, the main precursor of citrulline is glutamine, which is converted in the enterocytes of the proximal small bowel, accounting for 60%–80% of the total citrulline [6,25,26,27,28,29,30,31,32,33,34]. The small intestine releases the produced citrulline into the circulation, of which approximately 80% is taken up by the proximal tubular cells of the kidney for the arginine *de novo* synthesis [6,7,8,9,31]. This is also known as the intestinal-renal axis. The balance between gut synthesis and kidney degradation determines the plasma citrulline concentration [35]. Since the intestine is the main site of citrulline production, plasma citrulline concentration has been suggested as a biomarker of the functionality of the small bowel enterocyte mass [36,37,38], although the plasma concentration does not provide exact information concerning the balance between production and disposal. For example, renal failure is associated with an impairment of citrulline metabolism, because the kidney is the main organ that metabolizes citrulline into arginine [39,40,41].

In addition, citrulline can also be derived from the conversion of ornithine by ornithine transcarbamylase, which is present in the enterocytes and hepatocytes [42,43,44]. This produced citrulline is also exported and enters the portal circulation to bypass the liver and serve as arginine precursor. Furthermore, the dietary intake of arginine account for 40% of the circulating citrulline [42,43,44].

In the arginine *de novo* synthesis, ASS catalyzes the condensation of citrulline and aspartate to form argininosuccinate, the immediate precursor for arginine [24]. The produced argininosuccinate is split by ASL to release fumarate and arginine [45]. ASS and ASL are the rate-limiting enzymes for the intracellular arginine regeneration form citrulline [46,47,48,49].

The arginine *de novo* synthesis is not limited to the kidneys, although the kidney is the only organ that significantly releases newly synthesized arginine into the plasma in an amount to sustain whole-body arginine requirements [30]. The citrulline, not used in the kidneys, is transported via the systemic circulation for intracellular arginine *de novo* synthesis in several different cell types, such as macrophages [47,50], aortic smooth muscle cells [51], neural cells [52] and endothelial cells [46,53].

In the endothelial cells, ASS and ASL located in the caveola are part of the NOS3 complex, and their activity is essential for maintenance of a functional NOS3 complex, to maintain sufficient NO production [46,54] (Wijnands *et al.*, unpublished data).

The intracellular arginine *de novo* synthesis in macrophages does not play a role during physiological conditions, however in case of arginine starvation in inflammatory conditions this pathway may becomes essential [50,55], which will be discussed in more detail later in this review.

### 2.2. Arginine and Citrulline Transport Systems

The produced arginine is transported by specific arginine transport systems into the cells, to provide in the arginine demand of cells. The most important arginine transporter is y^+^ (for recent review [56,57,58]), which consists of four different types of cationic amino acids transporters (CATs); CAT-1, CAT-2a and CAT-2b, CAT-3 and CAT-4 [58,59]. Since CAT-2a, CAT-3 and CAT-4 have a very low affinity for cationic amino acids arginine and citrulline, only CAT-1 and CAT-2b will be discussed in this review.

CAT-1 is expressed in almost all adult cells [57], such as endothelial cells [59,60,61] and epithelial cells. In endothelial cells, CAT-1 is co-localized with NOS3 in the plasma membrane caveolae which facilitates specifically arginine channeling for the endothelial NO production [62]. This co-localization results in exclusive arginine availability for NOS3, preventing mixture of arginine in the intracellular pool [59,60,61]. CAT-2b is only expressed after induction with cytokines or lipopolysaccharide (LPS) treatment in inflammatory cells and is highly associated with NOS2 expression [59,63] and will be discussed in more detail in the next section of this review.

In basal conditions, arginine transport across the cell membrane of human monocytes [64] and murine bone marrow-derived macrophages [63,65] is mainly mediated through y^+^ associated with CAT-1, while after activation of the immune response, the increase in transport is due to CAT-2 [63,65].

Uptake from food-derived citrulline in the enterocytes of the small bowel is regulated by different transport systems [66], such as B^0,+^, L, and b^0,+^ systems [67]. Presence of system L in the cell membrane of intestinal epithelial cells allows passage of citrulline in both directions depending on the concentration gradient [68]. Similar to system L, transporter systems b^0,+^ and B^0,+^ are also localized in the cell membrane [67] regulating citrulline uptake. Also the main glutamine transporter in the intestine belongs to transport system B^0^, the SLC6A19 or B0AT1 transporter, which is located in the epithelial cell layer of the intestinal microvilli [69].

### 2.3. Catabolic Pathways of Arginine during Basal Conditions

Besides its dietary intake and endogenous production, arginine availability is also influenced by arginine clearance. Arginine catabolism can be catalyzed by five different group of enzymes; arginases (arginase-I and arginase-II) as part of the urea cycle, nitric oxide synthases (NOSs for the NO production), arginine decarboxylase (ADC) and arginine:glycine amidinotransferase [70]. Through these pathways arginine gives rise to ornithine, urea, polyamines, proline, NO and citrulline, proteins, glutamate, agmatine and finally creatine. In this review only the arginase and NOS pathways during basal and inflammatory conditions such as sepsis will be discussed (see [16,19,71,72,73] for additional reviews on these pathways).

### 2.4. Arginase Pathway: Synthesis of Urea, Ornithine, Polyamine and Proline

Approximately 15%–20% of arginine enters the urea cycle [11] where the enzyme arginase converts arginine into urea and ornithine [11,74]. There are two different isotypes of arginase; arginase-I and arginase-II. The cytosolic arginase-I is highly expressed constitutively in the liver, which accounts for 20% of the whole body conversion of arginine into ornithine [74,75]. Furthermore, arginase-I is also expressed in macrophages [65,76,77] and endothelial cells [76,78,79,80,81]. Mitochondrial arginase-II is expressed in low concentrations in extrahepatic tissue, such as kidney, brain, small intestine [82], mammary gland and macrophages [72,83,84,85]. Type II arginase is mainly involved in the syntheses of ornithine as precursor for proline and polyamines synthesis, such as spermine, spermidine, and putrescine [81,86].

In endothelial cells, both arginase isoforms are expressed constitutively, although the expression of the specific isoforms differs between the species [72,87]. Arginase activity can consequently regulate NO synthesis, promote the production of glutamate and proline due to expression of ornithine aminotransferase and enhance cellular polyamine levels [81]. In the presence of *arginase-I* overexpression in the small intestine, as present in transgenic mice, an impaired growth and development was observed as a result of a decreased arginine availability [88].

### 2.5. NOS Pathway (NOS1, NOS2, NOS3); Synthesis of Citrulline and NO

The nitric oxide synthase (NOS) enzymes convert arginine into NO and citrulline. The total production of NO depends on the activity of these NOS enzymes [89,90,91,92]. Three isoforms of NOS exist: neuronal NOS (NOS1 or nNOS), inducible NOS (NOS2 or iNOS) and endothelial NOS (NOS3 or eNOS) [93]. Generally, NOS1 and NOS3 are constitutive Ca^2+^ dependent enzymes that are expressed at low levels in various cell types [94]. The function of the NOS2 enzyme is not calcium-dependent [95] and is mainly expressed in macrophages and tissues in response to (pro)inflammatory mediators [50,94,96,97,98], this will be discussed in more detail in the next section of this review.

NOS1 is expressed in central and peripheral neurons [99] and other tissues and cell types such as the adrenal glands [100], the kidney [101], epithelial cells [101], smooth muscle cells [102,103] and pancreatic cells [104]. The function of NOS1 includes synaptic plasticity in the central nervous system, central regulation of blood pressure, smooth muscle relaxation [105,106], and vasodilatation via peripheral nitrergic nerves; nerves in which transmission is regulated by NO [101,103,107,108,109,110,111].

NOS3 is expressed in endothelial cells and regulates vascular tone [112,113,114,115,116] and exhibits vasoprotective effects such as preventing vasoconstriction, leucocyte adhesion and platelet aggregation [117,118] (see [119] for a recent review). Based on these vasoprotective effects and the regulation of vascular tone NOS3 is therefore considered as the main source of NO during basal conditions and endotoxemic conditions in the microcirculation [3,102]. As described above, NOS3 is co-localized with ASS and ASL in the caveola of endothelial cells and regulates the endothelial derived NO production via this pathway [3,54,61,119]. However, in the presence of a dysfunctional NOS3 complex, by tissue-specific ablation of the *Ass* gene, no alterations in blood pressure or vasomotor responses were observed in arteries of healthy mice, although the intracellular regeneration of arginine was diminished [120]. This indicates that arginine resynthesis is not rate-limiting for the NO production in the endothelium of healthy arteries, and only becomes important in case of a disease state or in case of a diminished availability of arginine.

The NOS enzymes require several cofactors for NO synthesis, of which tetrahydrobiopterin (BH4) is the most important one [121,122]. BH4 stabilizes the NOS structure and enhances the binding of l-arginine to NOS (see recent review [119]). Therefore, diminished availability or presence of BH4 results in NOS uncoupling, and results in superoxide production [122,123].

## 3. Arginine-NO Metabolism during Sepsis and Inflammatory Conditions

**Figure 2:**
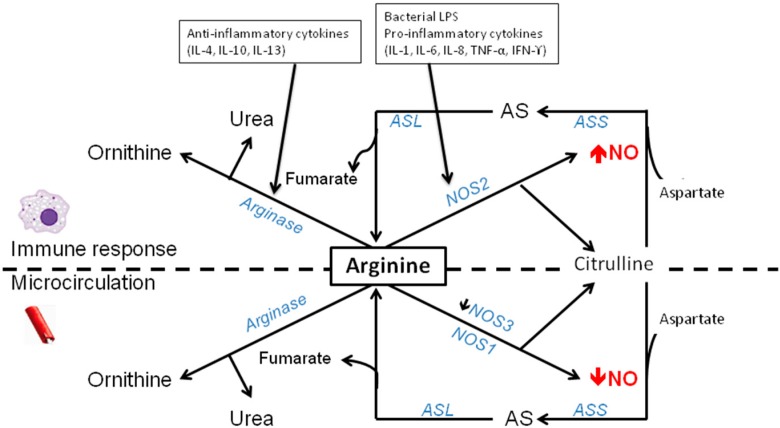
Arginine-citrulline-NO metabolism during inflammatory conditions. Abbreviations: NO, nitric oxide; NOS3, endothelial nitric oxide synthase; NOS2, inducible nitric oxide synthase; ASS, argininosuccinate synthase; ASL, argininosuccinate lyase; AS, argininosuccinate. Essential in developing multiple organ failure is the competition for arginine between the microcirculation and the inflammatory response. NOS3 is downregulated, while NOS2 is upregulated in response to pro-inflammatory cytokines. This will lead to more NOS2 induced NO production which is essential in the immune responses. Similar, arginase, mainly arginase I is upregulated by anti-inflammatory cytokines but is expressed in a later phase of infection and will therefore prevent NO overproduction by NOS2.

Sepsis and inflammatory conditions are characterized by organ dysfunction [124,125], as a result of a misdistribution of blood flow and low peripheral vascular resistance especially at the microcirculatory level which in turn results from the inflammation and endotoxemia [126,127,128]. Furthermore, sepsis is considered to be an arginine deficient state [2,3,4,129] with arginine becoming a semi-essential amino acid during stressed conditions such as sepsis [2,130] (see Figure 2). Although more arginine is being released from protein breakdown [131], it is also accompanied by an enhanced consumption of arginine, an impaired arginine *de novo* synthesis and a decreased supply of arginine, as observed in septic patients compared with healthy adults and with non-septic intensive care unit patients, leading to an impaired availability [1,2,131]. However, this does not necessarily mean that arginine deficiency occurs universally in all septic or inflammatory conditions. It is beyond the purpose of this review to determine whether arginine deficiency is present in all these conditions. Therefore, we focus on the available literature on arginine deficiency in human sepsis and animal models, using prolonged endotoxemia to mimic the arginine deficient state as observed in human septic patients. We previously observed decreased arginine concentrations in murine endotoxemia and in septic ICU patients [3,132]. During murine endotoxemia, arginine deficiency was present accompanied by a decreased NO production in the jejunal tissue of these animals. As a result, an impaired microcirculation in the jejunal villi was observed [3]. In the septic ICU patients, the decreased arginine concentrations were present independent of the cause of sepsis [132].

Several causes may be responsible for this impaired arginine production or the enhanced catabolism of arginine. The impaired arginine production can be the result of limited citrulline availability for arginine *de novo* synthesis, which may result from decreased nutritional intake in the critically ill patient [1,133,134,135,136], decreased uptake of protein by intestinal failure [2] or an impaired glutamine-to-citrulline conversion despite adequate splanchnic glutamine uptake [137]. Furthermore, renal failure can limit arginine *de novo* production from citrulline [39,40,41,138].

Arginine utilization is enhanced by the catabolic activity of arginase [2,139,140] and NOS2 or by an increased protein synthesis during inflammatory conditions [71,76,131,141,142,143,144]. In the past, an enhanced NO synthesis during the initial phase of sepsis was suggested. This was considered as observed by enhanced plasma concentrations of nitrate/nitrite (NOx) [145,146]. Based on these increased plasma nitrite and nitrate levels in septic patients, and on the fact that cytokine-induced NOS2 expression releases more NO during experimental conditions compared to the other NOS enzymes, a key role for NOS2 in the hemodynamic changes of sepsis was expected [147]. More recent studies indicate that the role of NOS2 expression rates and excessive NO production in causing hypotension is overrated [148,149], which will be discussed in more detail in Section 3.4. To estimate the NO production in septic patients, measurement of NOx levels was frequently used. As recently observed, increased plasma levels of NOx were observed in septic ICU patients compared to control patients [131,146]. These concentrations were even significantly increased in septic patients with hemodynamic failure or fatal outcome [146]. However, this enhanced nitrate/nitrite levels do not necessarily reflect an increased NO production during sepsis or endotoxemia, as several causes may result in these elevated plasma concentrations [2,150]. At first, a possible slower turnover of NOx in the plasma during sepsis may result in the increased concentrations of NOx, as observed in hypotensive septic patient [150]. A second contributor to higher plasma NOx concentrations may be the impaired renal excretion of NOx, as a result of renal failure as present in sepsis [151,152]. Another important explanation may be the discrepancy in time-specific changes, per hour and day, in the NOS enzyme activity during the course of endotoxemia and sepsis [153,154], which may result in an increased plasma nitrate concentrations as result of the delay in conversion into nitrate and the renal excretion [8]. Thus, the above-mentioned explanation has to be kept in mind when interpreting studies using NOx concentrations as marker for NO production.

Another method used to determine the NO production in sepsis is by using stable isotopes [2,131,150]. In recent years a number of research groups used this method to measure the *in vivo* whole-body NO production. Data from these studies indicate that the whole-body NO production was significantly reduced in sepsis compared to critically ill control patients and healthy subjects [2,131,150]. However, the possible compartmentalization of arginine [61], intracellular or within organs, may influence the NO production measurements with stable isotopes, as this is not used in the calculation of whole-body NO production [155]. As observed, this may also contribute to differences measured in NO concentration with NOx or total body NO production [2,131,150]. Furthermore, the location of the expressed NOS-isoforms within the organs may influence the measurement of the NO production. As described by Cauwels *et al.* [148], non-hematopoietic cells are essential for the systemic NO production during inflammation, such as the intestinal enterocytes, the Paneth cells or hepatocytes. Therefore, these different expression sites, which may be differently affected by inflammation and sepsis, can also lead to unparalleled concentrations of NO production. For example, nasal NO measurements is a good sensitive and specific biomarker for nosocomial infection acquisition in the upper airway and respiratory tract [156,157,158]. In patients with maxillary sinusitis and sepsis, nasal NO production was significantly reduced in the sinus epithelium compared to control patients, suggested to be the result of reduced NOS2 mRNA expression [157]. Also in patients with acute respiratory distress syndrome (ARDS) low NO levels were measured, which as suggested by the authors may be the result of the rapid reaction of NO with other molecular species, and therefore of less value to measure pulmonary inflammation [159]. As for patients with pneumonia on mechanical ventilation, the NO levels were significantly increased, although basal concentrations were significantly lower compared to other studies [151]. In line, in acute lung injury patients increased urine NO was measured, which was associated with a better outcome, which may reflect more perfused lung beds and associated decreased organ failure [160].

Another suggestion is the time point at which the NO production is measured which may influence the differences in NO production and outcome of the abovementioned studies. This time-point is influenced by the expression of the enzymes, the possible plateau phase of NOS expression or the maximum NO production [153,161]. This plateau phase of NO production further depends on sufficient substrate availability, in other words arginine availability and its co-factors [2,3,131]. As described in the clinical sepsis studies and experimental studies with endotoxemia, arginine deficiency is related to sepsis and also to a decreased NO production [2,3,129,131,146]. All the abovementioned factors may lead to the discrepancy between the low NO production measured in tissue or plasma during sepsis and the increase in nitrite/nitrate plasma or urine concentrations as measured in experimental and human sepsis. Thus, measurements of total NO production during sepsis and endotoxemia may only be an estimation of the total production or presence of NO in the tissues during sepsis and endotoxemia.

On the other hand, the production of NO by NOS3, especially for the endothelial cell, is decreased during sepsis and prolonged endotoxemia [3,162]. Decreased arginine availability is suggested to play a role in this downregulation of NOS3 and thereby in the decreased bioavailability of NO. This reduced NO results in endothelial dysfunction [163,164].

The immune response also contributes to the arginine depletion. During infection and inflammation, macrophages become active in response to a range of stimuli including damaged cells, pathogens and inflammatory cytokines such as TNF-α [165]. Non-activated macrophages exhibit minimal utilization of arginine and do not express NOS2 or arginase activity. Macrophages are classified based on their two main functions resulting in 2 phenotypes; the M1 macrophages which “fight and kill” and the M2 macrophages which “restore and heal tissue” [166]. As part of the immune response, M1 and M2 macrophages stimulate T-lymphocytes to produce Th1 or Th2 cytokines to maintain the predominant type of immune phenotype [166]. Upon activation by the immune response, M1-macrophages actively import extracellular arginine to synthesize NO by NOS2 [50]. This enhanced NOS2 induced NO production in peripheral blood mononuclear cells is likely to be associated with an increased arginine transport across the cell membrane [167]. Hypothetically, decreasing the arginine availability during sepsis may, therefore function as a protective mechanism to decrease excessive NO production by NOS2 and to regulate excessive adaptive (T-lymphocyte) immune responses, preventing possible excessive inflammation during sepsis. Arginase, expressed by M2-macrophages, which is part of the anti-inflammatory response, competes with NOS2 for arginine and therefore contributes to arginine deficiency for M1/Th1-type macrophages [133,168,169] (see [170] for an excellent review on the competing arginine pathways in macrophages). However, this not only results in less NO production by M1 macrophages, but also in an impaired T-cell function [133,169], which is not beneficial during sepsis. The capacity of intracellular arginine *de novo* synthesis in M1 macrophages partly preserves the arginine availability for NO synthesis [171], and as will be discussed in more detail below, also presences NOS2 derived NO synthesis, which is essential in the immune response as part of the host defense. As for the T cell function, arginase-induced arginine depletion results in suppression of T-cell activation, proliferation and differentiation *in vitro* [172,173] and *in vivo* [174,175,176]. However, in human T-cells, this arginine depletion does not seem to impair the important aspects of T-cell function such as chemotaxis and cytotoxicity [169]. Therefore, these findings, strengthen the believe that a decreased arginine availability may not be beneficial in sepsis, although a more balanced arginine availability may be essential, which preserves arginine for both pathways in the immune response, and so maintains the balance between NOS2 and arginase in inflammatory conditions.

As observed by our group, in tissue-specific arginase deficient mice, lacking arginase in hematopoietic cells, NOS2 produced significantly more NO during endotoxemia in the tissues and in macrophages compared to control [76]. However, this was not accompanied with an improved microcirculation in these animals, which may suggest the importance of compartmentalization of arginine. Based on the different expression levels and tissues in which the expressed NOS isoforms exist, this may lead to tissue specific differences in arginine and NO production, and therefore a disbalance in the local (micro)circulation and immune response. Thus, we believe sepsis and endotoxemia are not characterized by too much NO, but by a maldistribution of the produced NO, leading to perfusion differences and eventually organ dysfunction as a result of the impaired microcirculation.

### 3.1. Alterations in the Arginine and Citrulline Transport Systems in Sepsis

During sepsis or endotoxemia a shift in the expression of CAT transporters is observed as the increased cytokine production and bacterial endotoxins result in a downregulation of CAT-1 transporters and upregulation of CAT-2, mainly CAT-2b [56,58,167]. The upregulated CAT-2 is the main arginine transporter for activated macrophages [50,63,65,177], which import large amounts of extracellular arginine [63,65] for NOS2-derived NO synthesis [50,58,63]. CAT-2 is co-induced with NOS2 in these macrophages, which results in shifting arginine towards NOS2 instead of NOS3 [56], to maintain the required arginine availability for NOS2-derived NO production [178]. This shift in arginine transport is suggested to be a part of the immune host response to produce increased amounts of NO, as a response to endotoxemia and the bacterial load, to control pathogens [56,167]. In addition, LPS-induced macrophages produce the cytokine TNF-α during infection or septicaemia, which is capable of activation of other macrophages to participate in the host defence system [63,165].

### 3.2. Decreased Citrulline Availability as Cause for Diminished Arginine de novo Synthesis in Sepsis/Infection

Besides the observed decreased arginine concentration in sepsis and endotoxemia [2,3,4,129,132,179,180], endotoxemia and inflammatory conditions are characterized by a reduced citrulline production and bioavailability [2,131,181,182]. This reduced citrulline production and bioavailability may contribute to the decreased arginine *de novo* synthesis during sepsis and endotoxemia [2,3,131]. These low citrulline concentrations have been associated with higher mortality rates in this patient population [35,130,135,183]. Furthermore, lower citrulline concentrations are associated with decreased NO production [2]. Indeed, supplementation of citrulline during endotoxemia led to an enhanced NO production in tissues of these animals [3].

A number of mechanisms can be responsible for this reduced citrulline production. At first, a decreased nutritional intake may contribute to the observed low citrulline and arginine levels in the critically ill. Moreover, a reduced uptake of glutamine by the enterocyte as part of the intestinal failure [2,184] is suggested to contribute to the decreased citrulline synthesis [137], although normal levels of glutamine and ornithine have also been reported in these patients [136,183]. Impaired absorption of citrulline, arginine and glutamine as a result from a decreased blood flow in the intestinal villi may be a factor in this reduced uptake by the enterocyte [185]. Also an impaired glutamine-to-citrulline conversion plays a role in this citrulline deficiency [137].

As a compensation, ornithine transcarbamylase (OTC) may help to restore the citrulline availability during conditions with low protein intake, such as sepsis and endotoxemia [73]. OTC converts ornithine into citrulline, which can freely pass the liver to serve as a substrate for the arginine *de novo* synthesis in the kidney [186]. Also a reduction of protein synthesis and ureagenesis may aid in restoring the citrulline and arginine metabolism during these conditions [187].

### 3.3. Decreased Arginine Availability by Enhanced Arginase Activity in Sepsis

During inflammatory conditions, both arginase-I activity and arginase-II activity are induced [2] by specific cytokines [188] in macrophages and neutrophils [83,179,189] (see Figure 2). The cytokines that induce arginase activity are T-helper II produced cytokines, such as interleukins 4, 10 and 13, which preferentially induce the expression of arginase-I [77,190,191].

The expression of arginase occurs at different time points during the course of endotoxemia. As observed in an LPS-induced murine-endotoxemia model, arginase-I, already present in low levels before LPS induction, was upregulated in activated murine peritoneal macrophages at 12 h after the onset of endotoxemia. Arginase-I remained present during the total time course of the experiment with a peak expression at 36 h after the onset [142]. On the contrary, arginase-II exhibited an increase in expression after 3 h of LPS stimulation with a peak expression at 6 h after induction and slowly decreased afterwards [142].

As described above, arginase-I and NOS2 compete for the same substrate, which may suggest that the late onset of arginase-I expression is suggested to prevent the toxic effects of NOS2-induced overproduction of NO [83]. Indeed, the different time points of peak expression of NOS2 and arginase in murine endotoxemia models, after, respectively, 20 min, 4–6 h and 12 h for NOS2 [153] and 12–36 h for arginase may indicate this inhibitory effect [142,192,193,194]. Furthermore, the increased circulating neutrophils, with arginase containing granules [83] which are capable of releasing arginase in the extracellular space in case of cell damage or phagocytosis, further contribute to the depletion of extracellular arginine availability for NO synthesis during endotoxemia [133,143,179].

In endothelial cells both arginase-I and arginase-II are expressed during inflammatory conditions, which compete with NOS3 for arginine, resulting in an impaired NOS3-derived NO production and endothelial dysfunction [78,79,80,195,196]. We recently demonstrated that tissue-specific absence of arginase-I, in endothelial cells and macrophages, resulted in increased arginine availability. However, due to the increased NOS2 induced NO production this did not result in beneficial effects upon the microcirculation during endotoxemia [76]. In addition, an increased arginase-II activity in endothelial cells also resulted in enhanced arginine utilization. This led to limited substrate availability for NOS3; resulting in NOS3 uncoupling accompanied by a reduced NO production and augmented superoxide anions production [78,79,80].

### 3.4. Regulation of NOS2 during Sepsis/Inflammation

Historically, cytokine-induced NOS2 expression and consequential enhanced NO production were suggested to be key factors in the development of sepsis-induced acute hemodynamic changes [140,197,198,199,200] and end-organ damage [146,162,199,201]. The impact of NOS2 derived NO production was recently re-evaluated in an experimental LPS endotoxemia model indicating no association between NOS2 derived NO production and hypotension [148].

LPS and T-helper I cytokines, such as interleukin-1, 6 and 8, TNF and interferon-γ, can induce NOS2 expression as part of the cellular immune response [191,202,203,204]. On cellular level, sepsis results in a cytokine-mediated induction of NOS2 in almost any cell type [198], but especially in macrophages [197]. Other cell types, including hepatocytes [205], aortic smooth muscle cells [51], vascular smooth muscle cells [199,205,206], Paneth cells and enterocytes of the jejunum [148] also participate. As mentioned above, the non-hematopoietic cells contribute to the systemic NO production, and not the hematopoietic cells such as macrophages or endothelial cells [148,149].

This enhanced NOS2 expression plays a major role in the host defense mechanisms against various intracellular pathogens [197], and represents a major cytotoxic principle by accomplishing successful clearance and control of these intracellular pathogens [97,98]. Therefore, maintenance of NOS2 derived NO is essential during sepsis and endotoxemia as part of the host defense mechanism, as inhibition results in detrimental outcome, which will be discussed in Section 4.1.

### 3.5. Regulation and Expression of Constitutive NOS during Infection/Sepsis

During inflammatory conditions and especially during sepsis [207,208], NOS1 exhibits an important immunoregulatory role, involving both pro-inflammatory and anti-inflammatory pathways [209,210,211]. As part of the pro-inflammatory pathway, NOS1-induced NO production is suggested to act as a free radical and is prone to be converted into more reactive nitrogen species [209,212,213,214]. Endothelial cells express both NOS1 and NOS3, with NOS1 localized in the nucleus and NOS3 in the cytosol [215]. Previous studies already indicated an important role for NOS1 in the maintenance of the microvascularisation [102,105,106]. Furthermore, NOS1 localized in nucleus of endothelial cells, exhibits an anti-inflammatory role by preventing cytokine production after TNF stimulation in cultured endothelial cells [106,215,216].

As for NOS3, inflammatory conditions and the decreased arginine availability result in NOS3 uncoupling [48,119,217,218], endothelial dysfunction [219], with an increased superoxide production [119,220] and disturbed microcirculatory flow as a result [221,222,223].

Another important contributor to NOS3 uncoupling is the induction of asymmetric dimethylarginine (ADMA). ADMA and *N*^G^-methyl-l-arginine (l-NMMA) are the most powerful endogenous non-selective NOS inhibitors. ADMA competes with l-arginine for the active site of NOS and for y^+^-mediated uptake into cells (see [224] for recent review). These methylarginines are eliminated from the body by enzymatic degradation by dimethylaminohydrolase (DDAH) and renal excretion. Along with arginine availability, endogenous inhibitors of NOS, including ADMA, may affect NO synthesis [131]. During sepsis increased ADMA levels have been observed which inhibit NOS derived NO production resulting in an impaired microcirculation [225]. Furthermore, increased mortality in critically ill patients has also been related to elevated ADMA levels [226,227]. The correlation between arginine and ADMA, defined as the l-arginine/ADMA ratio, is decreased in adult sepsis patients [228] indicating a role of ADMA in the modulation of NOS-regulated microcirculation.

### 3.6. ASS during Sepsis/Inflammation

Inflammation results in an upregulation of ASS expression, especially in macrophages [229,230] and vascular smooth muscle cells [206,231]. This may indicate stimulation of the intracellular arginine *de novo* synthesis to maintain the NOS2-derived NO production [47,229]. During the early phase of inflammation and sepsis, with still sufficient extracellular arginine available, macrophages export approximately >98% of the intracellular produced citrulline into the plasma [20,50]. The intracellular regeneration of citrulline, therefore, only becomes important in case of arginine deficiency. During arginine deficiency, NO synthesis in macrophages depends on the import of citrulline to maintain the NOS2-induced NO production [50,232]. Since intracellular accumulation of citrulline is thought to facilitate excessive arginine *de novo* synthesis, which results in abundant amounts of NO, exporting citrulline is suggested to regulate the NO production in macrophages [47,233,234]. Another important role of ASS as part of the innate immune system’s defensive role, is the clearance and detoxification of LPS by binding to the active portion of LPS, lipid A, which result in inactivation of LPS [235,236]. In addition, ASS released from the liver into the systemic circulation neutralizes the extracellular LPS-induced cytotoxicity in response to inflammation [230].

Until know, no studies on ASL function during endotoxemia or inflammatory conditions have been conducted.

## 4. Modulation of Enzymes

### 4.1. Modulation of NOS

Historically, excessive NO production by NOS2 [237,238] was suggested to play an important role in the development of the key features of sepsis and endotoxemia, the systemic hypotension [239] and vascular hyporeactivity to vasopressor agents [240,241]. The inhibition of the excess NO formation by inhibiting NOS2 activity was therefore proposed as a treatment for endotoxic shock or sepsis in the past. Over the past years multiple studies, *in vitro* and *in vivo*, have been conducted to investigate the role of selective NOS2 inhibition or deficiency, all using different sepsis or endotoxemia models with different selective inhibitors, which resulted in inconclusive results as discussed in brief below [56,178].

Supplementation of the selective NOS2 inhibitor *N*-(3-(Aminomethyl)benzyl)acetamidine (1400W) [242], did not affect systemic hemodynamic parameters during prolonged hyperdynamic porcine endotoxemia [243], however was capable of attenuating the impaired intestinal oxygenation and energy state [162]. In another porcine endotoxemia model, the supplementation of the selective NOS2 inhibitor mercaptoethylguanadine (MEG) prevented hypotension and was capable of decreasing the amount of expired NO, however this did not result in an improved hepatosplanchnic metabolism [244].

*N*^ω^-nitro-l-arginine methyl ester (l-NAME) supplementation during porcine endotoxemia exhibited detrimental effects on the liver perfusion, as it reduced the flow even further during endotoxemia, while aminoethyl-isothiourea (AE-ITU) administration resulted in an improved liver blood flow [245].

Diminishing the arginine uptake for NOS2 in CAT-2 knock-out mice resulted in a reduced macrophage NOS2 activity compared to healthy control mice [56,178]. Furthermore, genetic ablation of NOS2 in mice exhibited a decrease mortality and enhanced microvasculature responsiveness in a cecal ligation and puncture induced sepsis model [199]. In addition, in the absence of NOS2 a decreased defense against bacterial inoculation was observed [246,247], which underlines that NOS2 also exhibits a protective role during endotoxemia. *In vitro* studies in rat macrophages and lung cells pre-treated with selective inhibition of NOS2, with AE-ITU and aminoguanidine (AG), resulted in significantly decreased NOS2 mRNA and protein expression [248,249,250]. The supplementation of the selective NOS2 inhibitor ONO-1714 in a septic lung injury model induced by cecal ligation and puncture in rats lead to a decreased NOS2 activity and improved survival. However, this beneficial effect was only present when ONO-1714 was administered 12 h after the onset of sepsis [208].

In an ovine acute long injury model, representing sepsis, which was induced by inhalation of *Pseudomonas aeruginosa* bacteria administration in the lungs, specific inhibition of NOS2 with AG did not prevent the characteristic hypotension during endotoxemia, and still exhibited a significant increase in nitrite production [251]. Also the investigation of a BBS-2, a more potent selective NOS2 inhibitor, was not capable of reversing the sepsis induced vasodilatation [207], which may indicate that NOS2 may not or only partially be involved in the vasodilatation during sepsis and inflammation. As recently observed in an experimental sepsis model with LPS, excessive NOS2-derived NO production may not be accompanied by hypotension or morbidity during inflammation [148]. This resulted in the usage of non-selective NOS-inhibitors, to determine the role of the other NOS enzymes in sepsis.

Supplementation of the non-selective NOS inhibitor l-NAME in an experimental hyperdynamic sepsis model in ewes lead to a normalized renal blood flow, indicating an important contribution of the local NOS1 and NOS3 derived NO production in the hypotension [252]. In a rat endotoxemia model, the beneficial and adverse effects of the selective NOS2 inhibitor AE-ITU and the non-selective NOS-inhibitor l-NMMA were studied to determine the influence on organ failure as caused by endotoxemia. Both AE-ITU and l-NMMA resulted in NOS2 inhibition and attenuated the liver dysfunction and circulatory failure in the liver as caused by endotoxemia [253].

However, non-selective NOS inhibition also exhibited unfavorable side effects, such as a decreased regional blood flow and accompanied decreased cardiac output as observed after the administration of l-NMMA or l-NAME [254,255]. Also a higher vascular resistance and increased mortality in LPS treated dogs was demonstrated after administration of the non-selective NOS inhibitor *N* omega-amino-l-arginine [256]. In line, prolonged inhibition of NOS by l-NAME, in patients with severe shock, exhibited an increased blood pressure and vascular resistance during the course of supplementation. However, the effect of l-NAME tended to decrease during the course of infusion, which led to the conclusion that l-NAME supplementation only exhibited minor effects on the enhanced mortality in the patients studied [257].

Furthermore, inhibition of NOS activity in patients suffering from sepsis using the non-specific NOS inhibitor l-*N*^G^-methylarginine-hydrocholine was associated with an increased mortality rate [258]. Patients supplemented with another non-selective NOS inhibitor, 546C88, exhibited a significantly higher mortality rate, as a result of increased cardiovascular deaths compared to placebo treated patients within the seven days follow-up [258]. In search of an explanation why this increased mortality was found, attention focused on the function of the constitutive expression NOS3 and NOS1. The NO that is produced under physiological conditions by these NOS isoforms regulates blood-flow distribution in organs. During inflammation and sepsis, this NOS1 and NOS3-mediated NO production is reduced [259], reducing blood flow to the individual organs, thereby compromising its function. Indeed, experimental evidence indicates that inhibition of NOS1 and NOS3 activity during sepsis increases hepatic and splanchnic damage [260]. Therefore, it is suggested that the upregulation of NOS2 during sepsis may compensate for the downregulation of NOS1 and NOS3 with respect to organ perfusion [179]. However, as observed in previous studies untimely or uncontrolled inhibition of non-selective NOS or selective NOS2 during septic shock may induce splanchnic ischemia seen in some experiments [261]. Indeed, early inhibition of NOS2 resulted in a trend toward decreased survival, whereas early inhibition of NOS1, by 7-nitroindazole infusion, exhibited an improved survival in a murine pulmonary sepsis model [262]. On the contrary, NOS1 inhibition during the early time course of this model, combined with a late NOS2 inhibition, exhibited potential beneficial effects by decreasing the oxidative stress [263]. Therefore, we believe that modulations in NOS enzymes activity during human sepsis need to be performed with great precautions based on the diverse outcome of the several experimental models and the previous detrimental outcome in human sepsis with NOS2 inhibition. The place at which and the time when the NO is produced may be the key to better outcome in sepsis and endotoxemia. Thus, maintenance of good NOS isoforms function, such as NOS3 in the microcirculation for tissue perfusion and NOS2 in the initial phase to fight the pathogens may be essential. However, this complex regulation mechanism of the arginine-NO metabolism remains to be investigated in future studies.

### 4.2. Modulation of Arginase during Inflammatory Conditions

Several methods are used to study inhibition of arginase activity, such as therapeutic intervention with the non-specific arginase inhibitors l-valine or l-norvaline, or by the usage of (tissue-specific) arginase deficient mice models. Supplementation of l-valine or l-norvaline resulted in arginase inhibition *in vitro*, which led to an increased NO production in bovine pulmonary cells treated with LPS [79]. This enhanced NO production was also observed after specific knock-down of arginase in an *in vitro* experiment in thoracic aortas of rats [196]. Since arginase competes with NOS for the same substrate, inhibition of arginase directly influences the NO production [79,139]. We recently demonstrated that tissue specific arginase-I deficiency in macrophages and endothelial cells exhibit an increased NOS2 mediated NO production in a prolonged murine endotoxemic model, which was abolished by NOS2 inhibition [76].

Arginase upregulation, as competitor for the substrate arginine, also contributes to endothelial dysfunction, by NOS3 uncoupling resulting in a decreased NO production [78,87,264]. As previous observed *in vivo* chronic inhibition of arginase by administration of 2(*S*)-amino-6-boronohexanoicacid restored the endothelial function in the vessels of the rats supplemented with the arginase inhibitor compared to control [80].

### 4.3. Modulation of ASS in Sepsis

Absence or reduction of ASS is detrimental due to a decreased NO production [55,206]. In conditions in which extracellular arginine is depleted from the environment, activated macrophages of ASS-deficient mice were not able to maintain its function as a fail-safe system to sustain NOS2-derived NO production [50]. Also in endothelial cells ASS exhibits an essential role as selective reduction of ASS protein levels by siRNA demonstrated a decreased cell viability and was accompanied by a 80% decrease in NO production in endothelial cells [46]. ASS deficient mice, as developed by Patejunas *et al*. [265] in 1994, resulted in animals with high levels of plasma citrulline, accompanied by hyperammonemia, which eventually lead to early dead within 24–48 h after birth [265]. Injection of a recombinant adenovirus carrying human *Ass* cDNA in these ASS deficient mice ameliorated this neonatal crisis, and resulted in an expanded life span of approximately 16 days [266].

In heterozygotic ASS deficient mice, ASS^+/−^ mice, significantly increased plasma citrulline and decreased tissue arginine concentrations were observed [267]. Furthermore, these results were accompanied by a decreased inflammatory response upon liver injury by pyrazole, as demonstrated by a decreased nitrosative stress, decreased TNF-α production, impaired apoptosis, and diminished neutrophil infiltration [267], which may be related to the role of ASS as part of the innate immune systems in the clearance and detoxification of LPS [235,236]. In our developed tissue-specific ASS deficient mice, with ASS deficiency in endothelial cells and macrophages [120], we observed similar results during prolonged endotoxemia in macrophages (Wijnands *et al.*, unpublished data). ASS deficient macrophages exhibited a significant decreased nitrite production upon LPS stimulation compared to littermates and also a decreased inflammatory response with decreased TNF-α production.

### 4.4. Modulation of ASL in Sepsis

The role of ASL modulations in sepsis has not been investigated thus far. However, recently ASL deficiency was studied in necrotizing enterocolitis, for which a enterocyte-specific heterozygotic deletion of ASL mouse model was developed (*Asl^flox/flox^*; *VillinCre^tg/+^* or CKO). This study showed that in the absence of ASL in the enterocytes an increased pro-inflammatory state and enhanced apoptosis occurred in these animals [268]. Another recent study, investigating ASL^−/−^ mice, showed early mortality within 48 h after birth in all homozygotes as a result of elevated plasma ammonia, argininosuccinic acid, glutamine, and citrulline concentrations to toxic concentrations [48]. In addition, significantly lower concentrations of arginine and nitrite production were observed in the ASL deficient animals, accompanied by an incompetent NOS3 complex formation, indicating the importance of intracellular regeneration of citrulline into arginine [48].

## 5. Enhancement of Arginine and Citrulline Availability in Sepsis and Inflammatory Conditions with Supplementation

### 5.1. Arginine Supplementation during Inflammatory Conditions

Over the past decades, supplementation of l-arginine was considered a logical therapeutic option to restore the decreased arginine levels in septic and critical ill patients, as increased arginine concentrations may restore the important physiologic processes, including organ perfusion, immune function, protein synthesis and wound healing [88,269]. Several studies have evaluated the effects of l-arginine supplementation in a variety of conditions, at first in healthy controls. Arginine supplemented in a dosage of three to eight grams a day, rarely resulted in unwanted side effects, suggesting the supplementation of arginine to be safe [270]. However, given in a higher dosage, exceeding nine grams, human subjects experience gastro-intestinal discomfort, vomiting and diarrhea. The severity of these side effects seem to be dosage dependent [12].

Previous meta-analysis and systematic reviews evaluated the value of enhanced nutrition as a possible additional treatment in surgical patients and critically ill patients. Most studies using arginine as supplement [271,272] investigated a combination of different nutrition compounds [134,273], such as omega-3-fatty-acids, glutamine [31,274,275], or nucleotides [275], unfortunately all with different results. A prospective, randomized, double-blind, placebo-controlled study in ICU patients, used oral arginine supplementation added to the enteral nutrition which resulted in enhanced arginine and ornithine concentrations, possibly by an increased arginase activity in this patient population without an enhancement of the NO production [276]. As for the clinical outcome, in a randomized control trial enteral formula enriched with arginine, and other select nutrients, showed no differences in hospital mortality, infectious complications or ICU length of stay between the arginine supplemented group and the placebo treated ICU patients [277]. In line, in another study in critically ill trauma patients [278], no differences in the arginine supplemented *versus* placebo treated group on clinical outcomes were observed. However, a study in severe sepsis patients has proven otherwise [279] as a significantly higher mortality was observed in the enteral supplemented patients with arginine containing diets [279]. Although all studies agree that enhancing the metabolic response in patients and especially the critical ill is necessary to improve outcome and to reduce mortality and complications [280,281,282,283], the different results of these studies led to the questionability of the effectiveness of supplementation, and in this case also of arginine supplementation [282]. Therefore, experts in the field suggested not to use arginine supplementation in critical ill patients, as this may result in an enhanced mortality rate [284,285]. However, this questionability and the assumption of an increased mortality especially after arginine supplementation in sepsis and critical ill patients may be confounded by grouping different formulas and different types of patients together. As previously observed in elective surgical patients, a significant reduction in infectious complications and a trend towards improved mortality in patients supplemented with nutrition containing high arginine concentrations was present [281], suggesting these negative results of arginine to be patient population and interventional driven [281]. Therefore, good-quality studies examining l-arginine monotherapy are essential to define the clinical usage of l-arginine in the critical ill patients.

Prior to the usage of l-arginine monotherapy in critical ill patients, our research group tested l-arginine monotherapy in experimental sepsis/endotoxemia models. l-arginine supplementation exhibited beneficial effects on the arginine concentrations without deleterious side effects in an endotoxemia model in pigs. Furthermore, in this model, pre-treatment with arginine prior to endotoxemia induction resulted in an increased hepatosplanchnic perfusion and enhanced NO production compared to untreated endotoxemic animals [75]. In addition, l-arginine supplementation not only enhances the immune response, but also the protein turnover in endotoxemic pigs [205] and resulted in elevated whole-body NO synthesis without adverse effects [286]. In line, in a murine endotoxemia model, l-arginine supplementation, starting during arginine deficiency, resulted in increased arginine plasma concentrations and an enhanced jejunal NO production without negative side effects [3]. These positive effects of l-arginine supplementation in experimental models led to the start of a new study of l-arginine supplementation in septic ICU patients.

The recent dose-titration study with l-arginine from our group showed increased plasma arginine concentrations, combined with an enhanced arginine *de novo* synthesis and increased NO production. More importantly, this did not result in negative alterations in hemodynamic parameters [180]. The absence of clinically adverse effects was also described in a previous conducted pilot study with l-arginine monotherapy in non-surgical critical ill patients [129]. In this study, l-arginine supplementation was associated with an increased ornithine synthesis, suggesting a preferential usage of the available arginine by arginase, with only a minimal enhancement of the citrulline/NO synthesis [129]. In line, the results from our endotoxemia murine model support this clinical observation, as the enhanced arginine plasma concentrations were not accompanied by enhanced intracellular arginine concentrations, but only with increased ornithine concentrations [3]. Thus, l-arginine monotherapy in septic patients seems to result in enhanced arginine concentrations and also increased NO production without adverse events. Therefore, based on beneficial effects without an adverse outcome, the issue of l-arginine supplementation during sepsis remains an actual point of discussion and to our opinion needs to be revisited.

### 5.2. Citrulline Supplementation during Inflammatory Conditions

Since reduced citrulline bioavailability in sepsis and endotoxemia, resulting in low plasma arginine [2,3] is associated with higher mortality rates [35,130,135], the supplementation of citrulline could be another therapeutic intervention restoring the balance between arginine production and metabolism as well as improving NO production and related functions. During endotoxemia and inflammatory conditions, supplementation of l-citrulline enhances plasma citrulline and arginine concentrations [3,17,287]. Oral supplementation of l-citrulline in endotoxemic rats resulted in higher plasma arginine concentrations compared to l-arginine supplementation [182], making l-citrulline a more efficient supplement than l-arginine during inflammation. We previously showed that l-citrulline supplementation resulted in an enhanced NO concentration and improved microcirculation during endotoxemia in the jejunal villi, which was not observed following l-arginine supplementation [3].

Study of Qualls and colleagues [50] showed no enhancement of NO synthesis under arginine overflowing conditions in LPS + IFN-γ stimulated rat fetal liver-derived macrophages after addition of citrulline. However, under conditions of arginine scarcity, addition of citrulline contributed to an enhanced NO production in that same model [50]. Interestingly, the effect of citrulline supplementation on increased NO production was only seen in arginine depleted macrophages, suggesting citrulline metabolism contributes to macrophage arginine biosynthesis and NO production only under conditions of arginine scarcity [50].

However, citrulline supplementation requires a functional NOS3 complex, and thus also a functional intracellular ASS-ASL complex. As previously demonstrated, in tissue specific arginase-deficient mice, an enhanced NOS2 pathway was present due to the absence of the inhibitory effects of arginase in the macrophage [76]. Citrulline supplementation in these animals did not lead to an improved outcome, based on the absence of a functional NOS3 complex.

## 6. Modulations in the Arginine-NO Metabolism

To illustrate the complexity and outcome of modulations in the arginine pathway, we added a table with an overview of our findings in murine endotoxemia, which underscores this complexity (see Table 1). As demonstrated, murine endotoxemia is characterized with arginine deficiency and also a decreased NO production in jejunal tissue of these animals. Furthermore, the microcirculation was also significantly decreased in these animals [3,76]. Since arginase-I and NOS2 compete for the same substrate, arginine, we developed a tissue-specific arginase-I deficient mouse model [76], to determine whether the absence of arginase-I in endothelial cells and macrophages would exhibit beneficial effects on the arginine concentration and NO production during endotoxemia [76]. The absence of arginase-I was accompanied by an increased inflammatory response and an enhanced NOS2 derived NO production. This increased NOS2 activity is suggested to contribute to the decreased NOS3 derived NO production and the depressed microcirculatory flow during endotoxemia. In addition, ADMA concentrations were significantly increased after prolonged endotoxemia in both mouse strains, which led to a significant decreased arginine/ADMA ratio. Since citrulline supplementation is suggested to be a more preferential substrate for NOS3 [3,61], to result in an enhanced arginine availability, we supplemented citrulline in control and tissue specific arginase-I-deficient mice during prolonged endotoxemia.

**Table 1:** Summary of modulations in the arginine-NO metabolism during endotoxemia to illustrate the complexity.

Outcome	Control			*Arg1^fl/fl^/Tie2-Cre^tg/−^*		
	Basal	LPS	LPS-Cit	Basal	LPS	LPS-Cit
Arginine	75	↓	↑/↑	↑	=/↑	↑/=/=
ADMA	0.5	↑	=/=	=	↑/=	=/=/=
Arginine/ADMA	127	↓	↑/↑	↑	↓/↑	=/↑/↑
NO production	6	↓	↑/↑	=	↑/↑	=/↓/↓
Microcirculation	401	↓	=/↑	=	↓/=	=/=/=

Results of wildtype (control) and arginase-I deficient mice (*Arg1^fl/fl^/Tie2-Cre^tg/−^*) during basal, LPS and LPS with citrulline supplementation. Arginine and ADMA concentrations are displayed in (μmol/L). The NO production is displayed in pmol MNIC/mg wet jejunal tissue per 30 min. The microcirculation is displayed in number of perfused vessels in jejunal tissue (*n*). Results from basal and LPS conditions were published previously [76]. The results are displayed as increase (↑), decrease (↓) or no effect (=). For control mice the results are displayed compared to basal in the LPS group. In the LPS-Cit group results were displayed as compared to control/compared to LPS. The results of the arginase-I deficient mice during basal conditions are compared to basal conditions of the control mice during basal conditions. During LPS results are compared to basal of arginase-I/compared to LPS of the control mice. Finally, for the LPS-Cit group results are displayed as compared to basal of arginase-I/compared to LPS of arginase-I/compared to LPS-Cit of control mice.

As expected, citrulline supplementation resulted in increased plasma arginine concentrations in control mice during endotoxemia. However, as for the arginase-I tissue specific deficient mice, arginine concentrations did not significantly increase compared to LPS-treated animals alone. Citrulline supplementation enhanced the NO production in jejunal tissue and the microcirculation in the jejunal villi. These results were not present in the arginase-I deficient mice, as the NO production and microcirculation in jejunal tissue did not improve after citrulline supplementation during endotoxemia. Furthermore, citrulline supplementation did not influence the ADMA concentrations in control or arginase-I deficient mice. However, the arginine/ADMA ratios were significantly improved in both mouse strains based on the effects on the arginine concentration during LPS and citrulline supplementation.

Based on these results, in arginase-I deficient mice, citrulline supplementation is not used as a source for NOS2 derived NO production, which may indicate that arginase-I deficient mice exhibit a dysfunctional NOS3-derived NO production.

These findings underscore the complexity of the interaction between substrate metabolism and the functional enzyme complex. Future research is essential in this field to unravel the complexity of the arginine-NO metabolism during endotoxemia and sepsis and most importantly to prevent arginine deficiency. At this moment, we are conducting a clinical study in septic ICU patients with citrulline supplementation to investigate whether citrulline is capable of enhancing the arginine availability and the microcirculation during sepsis in these patients.

## 7. Conclusions

In conclusion, arginine and its precursor citrulline play an important role in the immune response during inflammation and sepsis. Maintaining the arginine availability during inflammatory conditions is of crucial importance, probably best by enhancing the citrulline concentration, preventing NOS uncoupling and maintaining adequate enzyme function during these conditions. Future studies are warranted to determine the presence of arginine deficiency in other inflammatory conditions, the safety of supplementation and also to find the best supplement to enhance the arginine-NO pathway in these situations.
